# An Online Nutrition Education Program Targeting Intentions and Related Determinants Towards Dietary Supplement Use: An Application of the Theory of Planned Behavior

**DOI:** 10.3390/nu17030557

**Published:** 2025-01-31

**Authors:** Jana Daher, Margo Mountjoy, Dalia El Khoury

**Affiliations:** 1 Department of Family Relations and Applied Nutrition, University of Guelph, Guelph, ON N1G 2W1, Canada; jdaher@uoguelph.ca; 2 Department of Family Medicine, McMaster University, Hamilton, ON L8S 4L8, Canada; mountjm@mcmaster.ca

**Keywords:** theory of planned behavior, varsity athletes, dietary supplements, intervention, sports nutrition

## Abstract

**Background/Objectives:** Dietary supplement use among varsity athletes is influenced by various psychological and social factors, yet there is limited evidence on the effectiveness of educational interventions in influencing these determinants. The aim of this study was to determine the effects of an online nutrition education program on improvements in intentions and related determinants towards the use of dietary supplements in varsity athletes at the University of Guelph in Canada. The theory of planned behavior served as the theoretical framework for examining these determinants as predictors of behavior change. **Methods**: A randomized wait-list controlled trial was conducted on a total of 30 varsity athletes, randomized into experimental (n = 18) and control (n = 12) groups. The experimental group received access to an online nutrition education program focused on sports nutrition and dietary supplements, while the control group did not have access during the study. **Results**: There was a significant (*p* < 0.05) intervention effect on varsity athletes’ attitudes (Mean1_(control)_ = 13.17; Mean1_(experimental)_ = 13.56; Mean2_(control)_ = 13.92; Mean2_(experimental)_ = 12.11), perceived behavioral control (Mean1_(control)_ = 15.92; Mean1_(experimental)_ = 16.11; Mean2_(control)_ = 16.33; Mean2_(experimental)_ = 18.39), and intentions (Mean1_(control)_ = 12.5; Mean1_(experimental)_ = 12.89; Mean2_(control)_ = 11.58; Mean2_(experimental)_ = 9.44) towards dietary supplement use. No significant changes were made to descriptive and injunctive norms. **Conclusions**: These findings suggest that this nutrition education program significantly improved intentions and related determinants towards dietary supplement use in varsity athletes. The absence of a successful change in subjective norm should be a focus for similar future interventions.

## 1. Introduction

Changing deep-rooted human behavior is a notoriously complex process. Demographics, cultural background, personality, ideologies, and emotions are all factors that can influence human behavior [1]. Extensive research has been conducted to understand human actions, and many theories have been proposed to identify the determinants of behavior. The theory of planned behavior (TPB), first introduced by Icek Ajzen, is a promising social cognitive model that has been widely used to predict and explore behavior [2]. According to the theory of planned behavior, there are three main factors that can influence human behavior: attitudes, subjective norms, and perceived behavioral control (PBC) [2]. Attitudes are the favorable or unfavorable assessment of a certain action, usually guided by behavioral understanding about the likely results of performing a certain behavior [3]. Subjective norms, defined as the pressure exerted by a social circle to get involved in a specific behavior, are influenced by normative beliefs [4]. Perceived behavioral control, or the perceived readiness to act, is determined by underlying control beliefs, which can simplify or complicate performing a certain action [3]. Combining these three factors would lead to the formation of intentions, which are the direct predictors of behavior.

**Figure 1:**
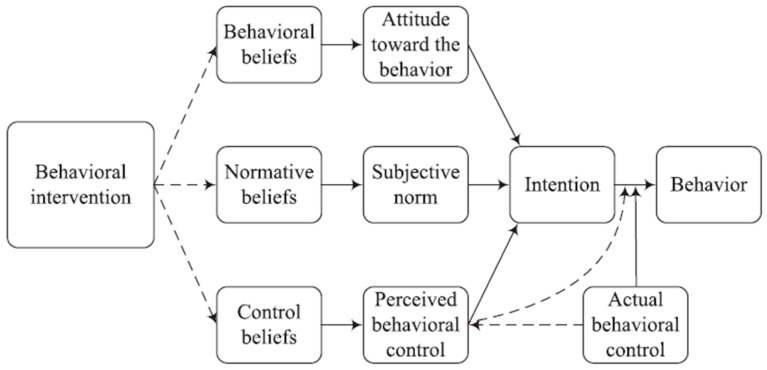
Expected effects of a behavioral intervention in the theory of planned behavior [2].

Despite the fact that the theory of planned behavior is better at predicting actions and behaviors than on assessing changes in behavior [5], it can serve as a conceptual framework to design effective interventions aiming to improve or change certain behaviors (Figure 1). A TPB-based intervention can improve subjects’ attitudes or supportive subjective norms, and elevate their perceived behavioral control, all of which can modify behavioral intentions and consequently predict behavior change [3]. Although interventions expose individuals to new information that can influence their beliefs, the importance of the three predictors as determinants of intentions varies based on the target behavior or the study population [2]. It is critical to identify if the underlying problem is due to a lack of motivation or failure to act on the existing favorable intentions before actually designing the TPB-based intervention. In case the intervention is established to create favorable intentions towards a certain behavior, it requires extensive pilot work and formative research to generate the motivation for change [3]. However, if the favorable intentions are already there, a different type of intervention is needed to close the intention–behavior gap that establishes ways to initiate and maintain the desired behavior [1].

While there is paucity of research on interventions around dietary supplements that adopt the theory of planned behavior, most of the available studies that explored the determinants and predictors of dietary supplement use have mainly focused on specific supplements, such as iron and vitamin D, multivitamins, and soy-based dietary supplements, within diverse populations that are difficult to compare. None of these studies have examined athletes as a target group [6,7,8,9,10,11,12]. A study by Alami et al. (2019) explored adolescent Iranian girls’ intentions to use iron and vitamin D supplements through the theory of planned behavior. The study found that TPB constructs, along with knowledge, accounted for 74% of the variance in behavioral intentions, with PBC and knowledge showing significant associations with supplement use intentions [6]. Another study investigated the predictors of use of multivitamin supplements by Caucasian college females and found that attitudes and perceived behavioral control are significant predictors of intentions to use multivitamins [11]. Ren et al. (2011) explored Chinese consumers’ intentions to use soy-based dietary supplements based on an integrated theory of planned behavior model, including soyfood favorability and dining-out sociability. Interestingly, the significant determinants of intentions were attitude, perceived behavioral control, and dine-out sociability, an important aspect of the Chinese local diet culture [12]. Athletes are a major part of dietary supplement users [13,14,15], which increases the concern of dietary supplement misuse, resulting in adverse health outcomes or in unintentional doping in this population. Therefore, it is critical to understand the psychosocial determinants and predictors of dietary supplement use among athletes, as well as how education interventions can effectively modify their behavior.

**Figure 2:**
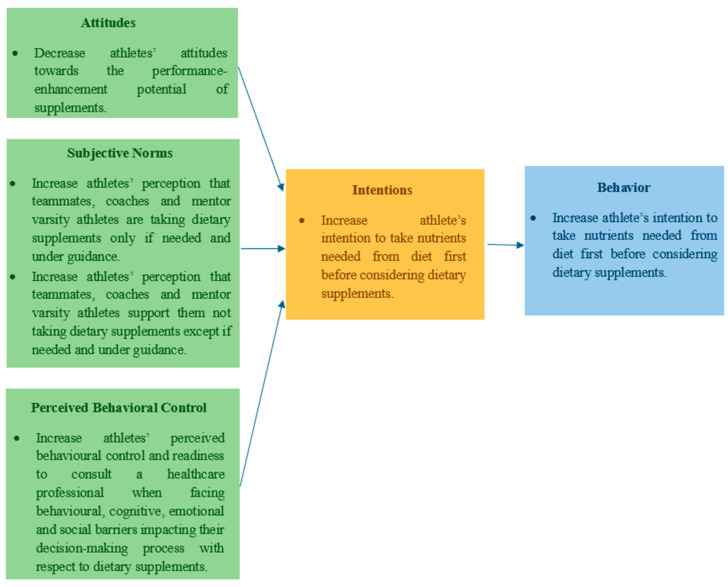
Objectives of the intervention based on the theory of planned behavior.

This study aimed at investigating the effectiveness of an online nutrition education program in influencing intentions and related determinants towards the use of dietary supplements in varsity athletes. The objectives of this study were to (1) decrease athletes’ attitudes towards the performance-enhancement potential of supplements, (2) increase athletes’ perception that teammates, coaches, and mentor varsity athletes are taking dietary supplements only if needed and under guidance (after diet alone has been considered), (3) and that teammates, coaches and mentor varsity athletes support them not taking dietary supplements except if needed and under guidance, (4) increase athletes’ perceived behavioral control and readiness to consult a healthcare professional (i.e., sports doctor, registered dietitian, etc.) when facing behavioral, cognitive, emotional, and social barriers that impact their decision-making process with respect to dietary supplements, and (5) increase athlete’s intention to take nutrients needed from diet first before considering dietary supplements (Figure 2).

## 2. Materials and Methods

### 2.1. Participants and Recruitment

The inclusion criteria were being a varsity athlete, a part-time or full-time student at the University of Guelph, and fluent in English. Thirty participants were randomized (simple randomization) into an intervention group (n = 18) and a wait-list control group (n = 12) as they were being recruited through an online randomization tool. The sample size was determined based on prior studies of a similar nature, which recruited comparable participant numbers [16,17]. After providing an electronic informed consent, participants in both groups were directed to fill out an online questionnaire. After that, the experimental group received access to an online nutrition education program for 4 weeks focused on sports nutrition and dietary supplements, while the control group did not have access during the study. After the 4-week period, the questionnaire was re-administered to all participants. Once the control group participants had completed the questionnaire, they were given access to the program for ethical reasons. As a token of appreciation for their time, participants received a CAD 50 gift card upon completion of the study. This research was approved by the University of Guelph Research Ethics Board (REB# 22-03-003).

### 2.2. Nutrition Education Program

**Figure 3:**
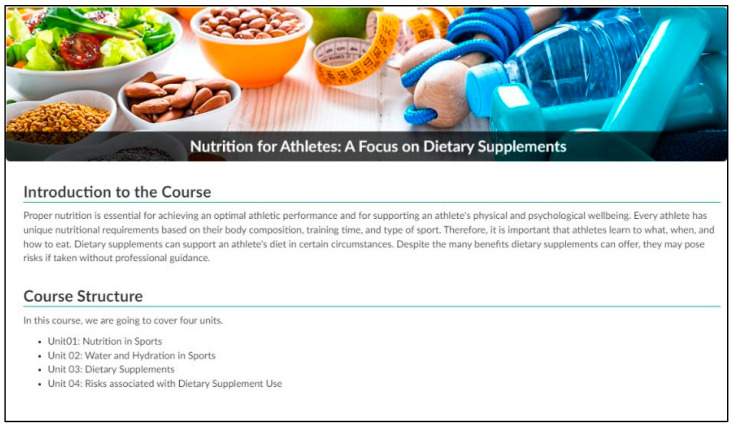
A snapshot from the “Nutrition for Athletes: A Focus on Dietary Supplements” Program.

The intervention consisted of an online nutrition education, titled “Nutrition for Athletes: A Focus on Dietary Supplements”. This program was published on CourseLink, the learning management system at the University of Guelph. It covered 4 units, with the following titles: (1) Nutrition in Sports, (2) Water and Hydration in Sports, (3) Dietary Supplements, and (4) Risks Associated with Dietary Supplement Use (Figure 3). Units were written in lay language, and included quizzes, myth busters, and fun facts. Each unit’s reading time is around 15–20 min.

The first unit, “Nutrition in Sports”, covered information on energy, carbohydrates, proteins, fats, and timing nutrition before, during, and after exercise. The second unit, “Water and Hydration”, covered information related to dehydration, ways to identify if you are dehydrated, and methods for preventing dehydration. The third unit on dietary supplements covered their definition, examples, and their uses, along with their categorization. The final unit, titled “Risks Associated with Dietary Supplement Use”, covered doping and fair play in sports, safety, quality, and the marketing of dietary supplements. It also discussed the risks associated with the use of dietary supplements and provided suggestions on how to use them safely. Participants were encouraged to complete one unit per week, but were allowed to progress at their own pace. The experimental group participants’ progress on CourseLink was tracked before the post-program questionnaire was administered. Participants who had not completed the four units received email reminders prior to being sent the post-program questionnaires.

### 2.3. Questionnaire Development and Design

The questionnaire utilized in this study was adapted from previous research and has been tested for validity and reliability on the target population [18]. Cronbach’s α values were 0.957 for intention, 0.542 for attitude, 0.831 for injunctive norm, 0.743 for perceived behavioral control, and 0.822 for descriptive norm. The validity of the questionnaire was confirmed through expert evaluations, achieving a median rating of 3 (“very relevant”). It included 2 major sections: (1) demographic information and (2) TPB constructs. It consisted of multiple-choice responses, Likert-based responses, and open-ended questions. The post-intervention questionnaire included an additional section seeking participants’ suggestions to improve the program.

The demographics section gathered information about participants’ age, gender, medical condition, parents’ or guardians’ education levels, ethnic background, smoking habits, and alcohol consumption while the theory of planned behavior constructs section included 20 statements about intention (n = 3), attitude (n = 4), injunctive norm (n = 5), descriptive norm (n = 4), and perceived behavioral control (n = 4). Statements in this section were rated on a five-point Likert scale, ranging from 1 (strongly disagree) to 5 (strongly agree) (Table 1). Examples of these statements are “In the next 6 months, I intend to take or keep taking a dietary supplement to improve my performance and/or general health (intention)”, “I believe that using dietary supplements will improve my performance (attitude)”, “I have complete control over whether to take or not to take dietary supplements from now on (perceived behavioral control)”, “My peers/friends think I should use dietary supplements to improve my performance, physical appearance or general health (injunctive norm)”, and “My immediate family (parents, brothers, sisters, grandparents, significant others) regularly uses dietary supplements to improve performance, physical appearance or general health (descriptive norm)”. The score range in Table 1 represents all possible scores a participant could achieve. For example, in the intention section, there were 3 statements. The minimum possible score for this section would be 3 (if a participant selected “strongly disagree” for all 3 statements, resulting in a score of 1 for each). The maximum possible score would be 15 (if the participant selected “strongly agree” for all 3 statements, resulting in a score of 5 for each).

### 2.4. Statistical Analysis

Statistical analyses were performed using the statistical software package for social sciences (SPSS) (version 24 for Windows), and statistical significance was determined at *p* < 0.05. Descriptive statistics were used for the participants’ demographic characteristics. A two-way mixed analysis of variance (ANOVA) was conducted following an assessment for assumptions. Outliers were assessed by examining studentized residuals for values greater than ±3. The normality of residuals was evaluated through Normal Q-Q Plots. Homogeneity of variances was assessed by Levene’s test of equality of error variances, and homogeneity of covariances was assessed by Box’s test of equality of covariance matrices.

## 3. Results

### 3.1. Participants’ Characteristics

Participants’ ages ranged from 18 to 30 years, with a mean age of 20.6 ± 2.9. The majority of participants were female, while males accounted for 30% of the sample. Most participants identified as Caucasian (67%), followed by Southeast Asian (10%) and West Asian (10%). None of the participants reported to be currently smoking, and approximately half reported to be currently consuming alcohol. The most common level of parents’/guardians’ education was a bachelor’s degree (43.3%) (Table 2). Participants were also asked about their training time per week. Overall, 47% trained for 11–15 h per week, while only 3% trained for more than 25 h per week.

### 3.2. Theory of Planned Behavior Constructs

Mean scores (±SD) and group x time effects for the TPB constructs are presented in Table 3.

#### 3.2.1. Intention

One of the key ANOVA results of this study was a statistically significant interaction between the intervention and time on intention to use dietary supplements, a direct predictor of behavior, F(1, 28) = 5.21, *p* = 0.030, and partial η^2^ = 0.157. This interaction effect suggests that the intervention was successful in decreasing mean scores of intentions in the experimental group, indicating a weaker intention of varsity athletes in this group to use or keep using dietary supplements to improve performance or health. Simple main effect analysis revealed that, for the experimental group, the intention subscale scores decreased from pre-intervention (M = 12.89, SE = 0.42, *p* = 0.001) to post-intervention (M = 9.45, SE = 0.86, *p* = 0.001).

#### 3.2.2. Attitude

**Figure 4:**
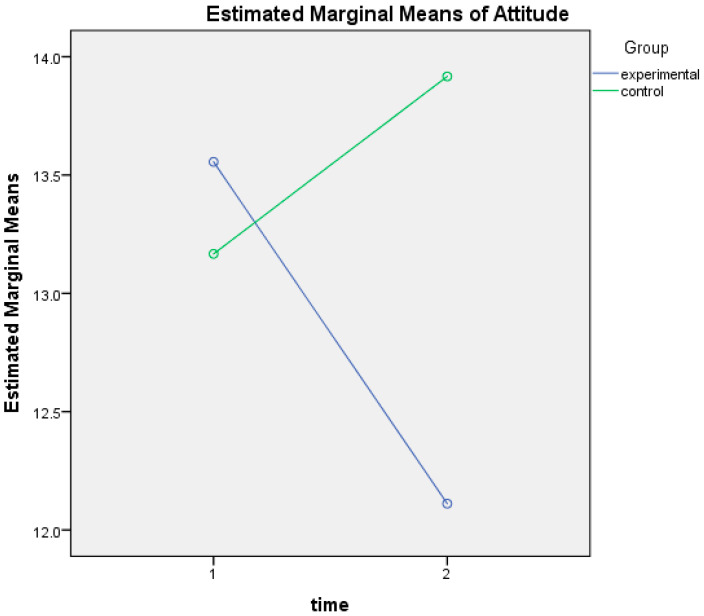
Change in attitude scores at baseline (time 1) and post-intervention (time 2) for control and experimental groups.

There was a statistically significant interaction between the intervention and time on attitude to use dietary supplements, F(1, 28) = 4.89, *p* = 0.035, and partial η^2^ = 0.149. Varsity athletes in the intervention group showed, over time, a decrease in positive attitudes towards the ability of dietary supplements to improve performance or health (Mean_(time1)_ = 13.56; SD_(time1)_ = 2.81; and Mean_(time2)_ = 12.11; SD_(time2)_ = 3.45), while athletes in the control group showed an opposite pattern over time, as their attitude scores increased (Mean_(time1)_ = 13.17; SD_(time1)_ = 2.98; and Mean_(time2)_ = 13.92; SD_(time2)_ = 3.20) (Figure 4).

#### 3.2.3. Injunctive Norm

There was no statistically significant interaction between the intervention and time on injunctive norm. However, the main effect of time showed that the injunctive norm subscale scores were statistically significantly higher at pre-intervention F(1, 28) = 4.2, *p* = 0.05, and partial η^2^ = 0.130 (Mean_(time1)_ = 15.71, SE = 0.85, *p* = 0.05) compared to post-intervention (Mean_(time2)_ = 14.33, SE = 0.92, *p* = 0.05). The main effect of group showed that there was no statistically significant difference in mean injunctive norm scores between groups.

#### 3.2.4. Descriptive Norm

There was no statistically significant interaction between the intervention and time on descriptive norm. Main effect analyses of group and time also showed no statistically significant differences.

#### 3.2.5. Perceived Behavioral Control

**Figure 5:**
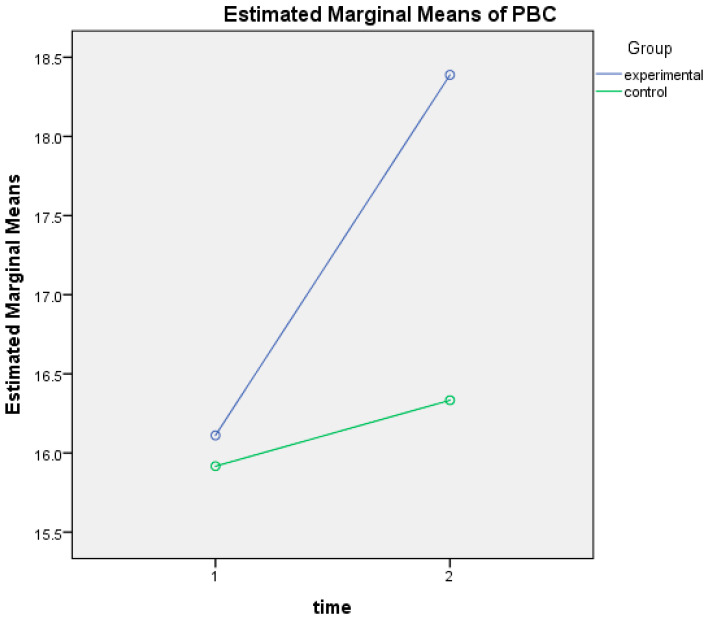
Change in perceived behavioral control scores at baseline (time 1) and post-intervention (time 2) for control and experimental groups.

There was a statistically significant interaction between the intervention and time on the perceived behavioral control towards using dietary supplements, F(1, 28) = 4.77, *p* = 0.038, and partial η^2^ = 0.145. After the intervention, participants in the experimental group exhibited a significantly stronger perception of the ability to control their decision to use or not use dietary supplements (Mean_(time2)_ = 18.39; SD_(time2)_ = 1.72), compared to the control group (Mean_(time2)_ = 16.33; SD_(time2)_ = 2.57), *p* = 0.014. PBC scores were statistically significantly higher after the intervention (Mean_(time2)_ = 18.39; SD_(time2)_ = 1.72) for the experimental group compared to pre-intervention (Mean_(time1)_ = 16.11; SD_(time1)_ = 2.06), *p* = 0.002 (Figure 5).

## 4. Discussion

This study assessed the effectiveness of an online nutrition education program in influencing intentions and related determinants towards the use of dietary supplements in varsity athletes. The theory of planned behavior was used as a theoretical framework to design and evaluate this study. Our findings indicated that the nutrition education program significantly improved varsity athletes’ intentions, attitudes, and perceived behavioral control towards dietary supplement use. However, the program did not produce significant changes in injunctive and descriptive norms. A large body of research has emphasized the capacity of attitudes, subjective norms, and perceived behavioral control in predicting health-related intentions and behaviors [6,7,10,11,12,19,20]. These studies were conducted on different populations and tested various health behaviors, mainly supplement use, which suggests the generalizability and applicability of the theory of planned behavior in predicting behavior change. However, the TPB constructs, depending on the nature of the behavior, would vary in terms of importance in predicting intentions and behavior change [21,22]. For instance, in situations where attitudes and PBC are strong, injunctive norms may be less predictive of intentions.

In our study, there was a significant intervention effect on attitudes, which refer to an individual’s positive or negative evaluation of performing a particular behavior [21]. A more favorable attitude towards a behavior typically leads to a stronger intention to engage in it. Consumers who think that dietary supplements can improve their health (i.e., have positive attitudes towards dietary supplements) have greater intentions to consume dietary supplements [9,23]. Our online nutrition education program was successful in decreasing the experimental group’s participants’ positive attitudes towards the ability of dietary supplements to improve performance or health. This finding is attributed to the interventions’ focus on addressing the behavioral beliefs of athletes about dietary supplements, not only by attacking existing beliefs, but also by introducing new information that prompts the formation of new beliefs. Attitudes are formed based on the corresponding set of behavioral beliefs, which is the information that people have about a behavior and its likely consequences [24]. Providing information that changes some of those beliefs or introduces new beliefs will likely lead to a change in attitudes to ultimately guide behaviors [24]. This concept highlights the role of education about the risks and benefits of a certain behavior in knowledge change and ultimately attitude formation/change [25]. Our nutrition education program placed a significant emphasis on the risks associated with dietary supplement use, which are often unknown to varsity athletes, specifically those who use dietary supplements [23,26]. This curricular emphasis may explain the underlying reason for a change in attitude among the experimental group participants.

While the insignificant effects on descriptive and injunctive norms (subcategories of subjective norms) are not surprising due to the nature of the intervention, it is important to note that both sub-constructs usually vary in terms of their significance in predicting behavior. Descriptive norms are based on the belief that the individual’s important others (e.g., friends, family, trainers, coworkers, etc.) perform a certain behavior, while injunctive norms are expectations that this circle approves or disapproves them performing this behavior [27]. Both beliefs contribute to the overall perceived social pressure to engage in a certain behavior. If an individual perceives that their significant others approve this behavior, they are more likely to intend to engage in it [22]. Several studies have found that injunctive norm is a stronger predictor of intention compared to descriptive norm, which suggests that the prevalence of supplementation among an athlete’s social circle is less likely to impact their supplementation behavior compared to the belief held by this circle about consuming dietary supplements [23,28]. A meta-analysis suggested that descriptive norms are usually better predictors of risk behaviors, whereas dietary supplement use is considered a health-promoting action [29]. This does not deny the fact that the intervention did not specifically address modifying athletes’ perceptions of social pressure towards dietary supplement use; however, it may have been successful in increasing their capacity to overcome this pressure, reflected by a significant increase in their perceived behavioral control and a weaker intention to use or keep using dietary supplements to improve performance or health.

Perceived behavioral control refers to the individual’s perception regarding the ease or difficulty of performing the target behavior [21]. It was added to allow the prediction of behaviors that are not completely under volitional control, which explains why intentions do not always predict behavior [22]. The degree to which behavioral control moderates the effect of intention on behavior is dependent on an individual’s ability to overcome barriers that may prevent them from acting on their intentions [27]. Although intentions are an immediate antecedent of behavior in the TPB, the mere formation of an intention is insufficient to predict behavior, especially when there is no complete volitional control over a certain behavior [22]. Therefore, perceived behavioral control becomes increasingly powerful as volitional control decreases, and may become independently predictive of behavior [21,22]. Interestingly, a study that investigated why women use dietary supplements found that intentions were a significant predictor of supplement use while perceived behavioral control was unrelated to the behavior [7]. Other studies on dietary supplement use found both constructs to be predictive of this behavior [6,10,23].

The limitations of this study are inherent to its nature. This study was based on self-reported data, which are susceptible to recall bias and social desirability. Secondly, the intervention was implemented in a university setting and focused on varsity athletes, which limits its generalizability to other sport-related contexts. Additionally, future research could benefit from a larger sample size. Finally, because the majority of participants were females, the study was unable to explore potential sex-based differences.

## 5. Conclusions

The results of this study indicate that an online nutrition education program can significantly improve varsity athletes’ intentions and related determinants towards dietary supplement use. This supports the need for implementing similar programs across educational institutions to guide their dietary behavior and encourage a food-first approach. Future studies can benefit from including other universities to examine the extent of application of the program and to have greater confidence in external validity. They should also aim to address subjective norms by incorporating peer discussions or integrating social influence components to shift perceptions around dietary supplement use.

## Figures and Tables

**Table 1:** Subscale interpretations and score ranges for the TPB constructs.

Construct	Low Subscale Meaning	High Subscale Meaning	Score Range
Intention	Weaker intention to use/keep using dietary supplements to improve performance or health	Stronger intention to use/keep using dietary supplements to improve performance or health	3–15
Attitude	Negative attitude towards the ability of dietary supplements to improve performance or health	Positive attitude towards the ability of dietary supplements to improve performance or health	4–20
Injunctive norm	Weaker perception that immediate family members approve their use of dietary supplements	Stronger perception that immediate family members approve their use of dietary supplements	5–25
Descriptive norm	Weaker perception that immediate family members use dietary supplements to improve performance, appearance, or general health	Stronger perception that immediate family members use dietary supplements to improve performance, appearance, or general health	4–20
Perceived behavioral control	Perceived difficulty in controlling the decision to use or not use dietary supplements	Strong perception of the ability to control the decision to use or not use dietary supplements	4–20

**Table 2:** Socio-demographic characteristics of participants.

Characteristics	Prevalence
*Participant Age (n = 30)*	
Mean ± SD	20.6 ± 2.9
Range	18–30
*Gender*	
Male	30%
Female	70%
*Ethnicity*	
Caucasian	66.7%
Southeast Asian	10%
West Asian	10%
Indigenous	3.4%
Latin	3.3%
Arab	3.3%
Prefer not to disclose	3.3%
*Smoking*	
Yes	0%
No	100%
*Alcohol Consumption*	
Yes	53.4%
No	43.3%
Prefer not to disclose	3.3%
*Parents’/Guardians’ Level of Education*	
University diploma above bachelor’s degree	36.7%
Bachelor’s degree	43.3%
College certificate or diploma	16.7%
High school diploma or equivalent	3.3%

**Table 3:** Mean scores, interactions, and simple main effects for TPB constructs.

TPB Constructs	Group	Pre-Intervention Mean (±SD)	Post-Intervention Mean (±SD)	Effect Group × Time		Simple Main Effect (*p*-Value)			
				*p*-Value	Partial Eta Squared	Group		Time	
						Pre	Post	Control	Experimental
**Intention**	Control	12.5 (2.54)	11.58 (3.53)	0.03	0.157	0.625	0.122	0.119	0.001
	Experimental	12.89 (1.78)	9.44 (3.65)						
**Attitude**	Control	13.17 (2.98)	13.92 (3.20)	0.035	0.149	0.72	0.16	0.202	0.061
	Experimental	13.56 (2.81)	12.11 (3.45)						
**Injunctive norm**	Control	15.92 (4.62)	13.83 (4.47)	0.30	0.038				
	Experimental	15.5 (4.49)	14.83 (5.18)						
**Descriptive norm**	Control	13.67 (2.35)	13 (2.48)	0.064	0.117				
	Experimental	11.72 (4.69)	13.94 (3.13)						
**Perceived behavioral control**	Control	15.92 (2.19)	16.33 (2.57)	0.038	0.145	0.807	0.014	0.339	0.002
	Experimental	16.11 (2.06)	18.39 (1.72)						

## Data Availability

The original contributions presented in the study are included in the article. The questionnaire is available upon request from the corresponding author.
